# Mid-Upper Arm Circumference (MUAC) shows strong geographical variations in children with edema: results from 2277 surveys in 55 countries 

**DOI:** 10.1186/s13690-018-0290-4

**Published:** 2018-08-15

**Authors:** Jose Luis Alvarez, Nicky Dent, L. Browne, Mark Myatt, André Briend

**Affiliations:** 1 International Rescue Committee, 3 Bloomsbury Pl, Bloomsbury, London, WC1A 2QL UK; 2 Preveyzieu , 01300 Contrevoz, France; 3 Department of Public Health, NHS Lothian, Edinburgh, UK; 4 Brixton Health , Llawryglyn, Wales SY17 5RJ UK; 5 Department of Nutrition, Exercise and Sports, Faculty of Science, University of Copenhagen, Frederiksberg, Denmark; 6 Center for Child Health Research, University of Tampere School of Medicine and Tampere University Hospital, Tampere, Finland

**Keywords:** Malnutrition, Community management, Edema, MUAC, Kwashiorkor

## Abstract

**Background:**

Severe acute malnutrition (SAM) is defined by a mid-upper arm circumference (MUAC) less than 115 mm or a weight-for-height z-score (WHZ) less than − 3 but also by the presence of bilateral pitting edema, also known as kwashiorkor or edematous malnutrition. Although edematous malnutrition is a life threatening condition, it has not been prioritized in recent research and has been neglected in global health initiatives.

**Methods:**

Two thousand two hundred and seventy-seven survey datasets were collected, and the age, sex, weight, height, MUAC and the presence or absence of edema were analyzed for more than 1.7 million children of 6–59 months from 55 countries, covering the period of 1992 to 2015.

**Results:**

During the last 10 years, the prevalence of nutritional edema was estimated at less than 1% in most of the countries where data were available. Some countries in Central and South Africa, as well as Haiti in the Caribbean, reported higher prevalence, and Yemen, Zimbabwe and the Democratic Republic of Congo reported prevalence between 1 and 2%. Surveys from a significant number of countries in Africa indicated that more than a third of SAM cases defined by MUAC < 115 mm had edema, including Malawi, Rwanda, Zambia, Togo and Cameroon. Children with edema were consistently shown across various analyses to have a significantly lower median MUAC than children without edema. However, the MUAC distribution had a large spread, with many children with edema having a MUAC > 115 mm, and this varied widely between countries, with median MUAC in edematous children ranging from 102 mm (Mali) to 162 mm (Sri Lanka). The proportion of SAM children with edema was found to be higher for older children.

**Conclusions:**

This study provides the most recent geographical distribution of nutritional edema and demonstrates that edema is a common manifestation of SAM, mainly occurring in Central Africa. The associated nutritional status, as assessed by MUAC, shows strong variation among children with edema. A more systematic and standardized system is required to collect data on edema in order to facilitate prevention, screening, referral and treatment of edematous malnutrition.

## Background

Severe acute malnutrition (SAM) is currently defined by the World Health Organization (WHO) and the United Nations Children’s Fund (UNICEF) by a mid-upper arm circumference (MUAC) less than 115 mm or by a weight-for-height z-score (WHZ) less than − 3 or by the presence of bilateral pitting edema [1].

The WHO ICD 10 classification [2, 3] defines kwashiorkor as a “form of severe malnutrition with nutritional edema with dyspigmentation of skin and hair.” The terms kwashiorkor and edematous malnutrition are often used for forms of SAM associated with bilateral pitting edema (referred to as “edema” in this article), without necessarily including associated dyspigmentation of skin and hair or any other clinical signs [4]. The latter meaning was chosen for this article because it is widely used in the field, especially in nutritional anthropometry surveys and for admission to therapeutic feeding programs.

Edematous malnutrition affects hundreds of thousands of children every year in the poorest countries of the world, resulting in high mortality [5], but the condition has not received much attention either in past years or in current research studies [6]. Given the high mortality risk associated with edematous malnutrition and the low level of understanding about the pathophysiology of edema [7], more research in this area is crucial. There is no mention of edema in the comprehensive implementation plan on maternal, infant and young child nutrition adopted by the 2012 World Health Assembly, which sets global nutrition targets for 2025. The condition is also overlooked in the 2013 “Maternal and Child Nutrition” Lancet series [8], which does not acknowledge its importance in public health terms or mention the existence of effective treatment when it calculates that 435,000 deaths could be prevented with the management of acute malnutrition every year. It has also not been mentioned in the most recent Global Nutrition Reports [9–12].

Currently, there is no reliable estimate of the prevalence or number of children suffering from edema in each country. Edema is not included in the Joint Child Malnutrition Estimates compiled by UNICEF, the World Health Organization and the World Bank [13], despite it being an independent diagnostic and admission criterion to therapeutic feeding services. Similarly, cases of edema are seldom identified and documented in standard national surveys, such as Demographic and Health Surveys (DHS) and Multiple Indicator Cluster Surveys (MICS). However, in recent years, some governments have undertaken national SMART (Standardized Monitoring and Assessment of Relief and Transitions) surveys during years when DHS and MICS have not been performed. SMART surveys include data collection on the presence or absence of edema, but often do not report on the prevalence of edema separately from Global Acute Malnutrition (GAM) and SAM by MUAC or WHZ [14].

To address a major gap in the knowledge base, this study investigated the distribution of edema in 55 different countries. In addition, the MUAC levels of edematous children were analyzed to better describe the relationship between wasting (MUAC < 115 mm) and edema, the two main clinical manifestations of acute malnutrition.

## Methods

### Description of surveys

Analyses were performed on 2277 survey datasets with information on 1,725,086 children aged from 6 to 59 months across 55 countries. Surveys were collected from 15 national governments/UNICEF country offices and 11 non-governmental organizations (NGOs), the Famine Early Warning Systems Network (FEWS NET), the United Nations Refugee agency (UNHCR) and the Food and Agriculture Organization (FAO) Food Security and Nutrition Analysis Unit (FSNAU) during the “Putting Child Kwashiorkor on the Map” initiative from January to September 2015. Signed agreements were made between NGOs and Action Contre la Faim (ACF) to analyze the data, and written permissions were gained from national governments via UNICEF.

Data was collected using mostly the SMART survey method [15], and all of the surveys followed a standard procedure. Most used a modified EPI two-stage cluster sample design, while some surveys from camp settings used a systematic household sample. The datasets included variables of interest for the study, which for each child were: age (months), sex, weight (kg), height (cm), MUAC (mm) and presence of edema. One MICS survey that collected MUAC and edema, in addition to other anthropometric measurements, was also included.

### Data cleaning

**Fig. 1:**
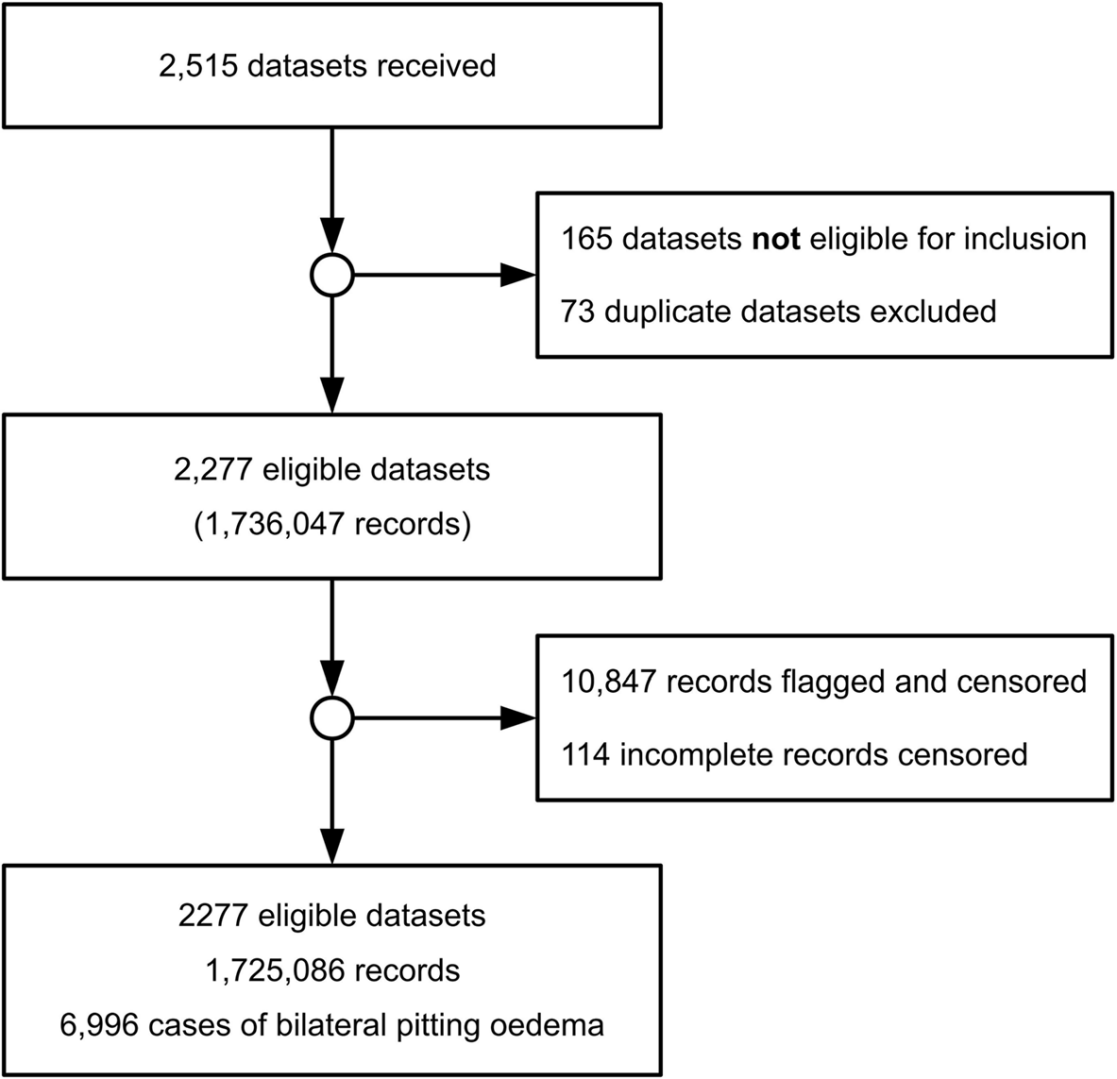
Selection of surveys for analyses

Figure 1 and Table 1 show the process of survey selection and data cleaning, including the number of received, ineligible, duplicate and eligible datasets, as well as the number of records flagged using the WHO flagging criteria, incomplete records and full records. Surveys were excluded if any of the key variables, except the cluster identifier, were missing. Possible duplicate datasets were detected using a simple checksum algorithm. These were confirmed using record-by-record validation. Confirmed duplicate datasets were removed from the database. Only variables of interest were selected to be included in the datasets. To standardize the datasets for analysis, variables were recoded and/or measurement units transformed into common codes and units.

**Table 1:** Data cleaning procedures for each variable in the database

Variable – Individual level	Issue encountered	Action taken	Rationale
Cluster identifier	Cluster identifier not specified	Retain record	Submitted surveys used systematic or simple random sampling
Age (months)	Children < 6 months or > 59 months; Age not specified	Record not used	6–59 months is the standard population in which prevalence of anthropometric deficits are reported in DHS, MICS, and SMART surveys
Sex (M/F)	Sex not specified	Record not used	Sex is required for determination of SAM status by WHZ
Sex (Other/M/F)	Sex unknown	Record not used	Sex is required for determination of SAM status by WHZ
Weight (kg)	Weight not specified	Record not used	Weight is required for determination of SAM status by WHZ
Weight (kg)	Weight obviously erroneous	Delete erroneous weight recorded	Data entry error
Height (cm)	Height not specified	Record not used	Height is required for determination of SAM status by WHZ
Height (cm)	Height obviously erroneous	Delete erroneous height recorded	Data entry error
MUAC (mm)	MUAC not specified	Record not used	MUAC is required for determination of SAM status
MUAC (mm)	MUAC obviously erroneous (< 60 or > 200)	Delete erroneous MUAC recorded	Data entry error
Edema (Y/N)	Edema and edema grade not specified	Record not used	Edema is required for determination of SAM status
Edema (Grade > 0/0)	If grade of edema recorded as > 0 instead of Y (Edema present) and grade of edema recorded as 0 instead of N (Edema absent)	Change grades > 0 to Y (Edema present) and grade 0 to N (Edema absent)	Study only takes into consideration presence of edema, regardless of grade
Edema (Other/Y/N)	If other unknown option recorded	Delete erroneous edema recorded	Data entry error

The data cleaning involved weight, height and sex which were not needed for this specific analysis but would allow further analysis involving WHZ. They were all incorporated into a database that will allow future investigation of the association between wasting and stunting. WHZ and edema analyses are not presented in this article, since, due to the mass of retained fluid, WHZ is greatly influenced by edema and interpretation of its geographical distribution would not be straightforward. Also, most decentralised outpatient services now use the MUAC definition of SAM as recommended by the Council for Research and Technical Advice on Acute Malnutrition (CORTASAM) [16].

Anthropometric indices were calculated using the WHO Child Growth Standards [1]. Data for individual children were flagged and excluded from the analyses but kept in the database. WHO flagging criteria were used to identify and remove records with outlying indices, but only for the analyses which concerned that specific index. Age was limited to 6 to 59 months, the standard survey population for most SMART-type surveys. Any obvious data entry errors were fixed or deleted.

### Merging the surveys

The cleaned datasets were stored in comma-separated value files. Metadata (i.e. survey dataset filename, location of survey, date of survey, and source of data) were stored in a separate comma-separated value file.

A plain text database was implemented. The R-AnalyticFlow scientific workflow software was used to organize, manage and analyze the database. R scripts were written to import the datasets, calculate anthropometric indices, implement flagging criteria for extreme values, determine datasets with abnormal results for edema (i.e. outlier surveys) and check for data quality and identify potential duplicate datasets. Given that only one survey was classified as an outlier, it was decided to include all surveys in the analysis.

### Data analysis

A set of purpose-written R language scripts, also managed using R-AnalyticFlow, were used to set inclusion criteria for data analysis, analyze the data, generate maps and report relevant results. Individual indicators were calculated for each country and each year to look for distributions and trends. Edema prevalence was calculated as the percentage of all children that were diagnosed with edematous malnutrition among the surveys. The proportion of SAM cases with edema was calculated as the children with edema among those with MUAC < 115 mm or edema. Comparisons of MUAC levels between children with and without edema, by age group and by sex, were done through the median of the distributions with 95% intervals and the Chi-Square test for trend.

## Results

A total of 2515 survey datasets were collected for 55 countries, spanning 1992 to 2015. 2277 non-duplicate datasets with 1,725,086 individual complete records were available for the final analysis (See Fig. 1).

Table 2 shows where surveys were conducted and the sample sizes of the data available in each country grouped by WHO member states’ regions. Most surveys came from the African region (74.3%), while only 3 European and 4 American countries were represented.

**Table 2:** Surveys analysed and sample size by region and country

WHO region	Number of countries	Country (Number of datasets; Number of children)
African	33	Angola (22; 17,361), Benin (7; 7930), Botswana (1, 164), Burkina Faso (50; 40,446), Burundi (25; 14,742), Cameroon (9, 5642), Central African Republic (58, 36,443), Chad (201, 124,096), Congo (Kinshasa) (264, 227,390), Cote d’Ivoire (49, 24,233), Eritrea (3, 1969), Ethiopia (233, 155,494), Gambia (8, 6769), Guinea (12, 9603), Guinea-Bissau (13, 7216), Kenya (107, 71,475), Liberia (52, 31,230), Madagascar (4, 3180), Malawi (16, 16,277), Mali (14, 10,968), Mauritania (56, 36,432), Mozambique (11, 3867), Niger (38, 49,411), Nigeria (107, 66,398), Rwanda (21, 13,534), Senegal (7, 8445), Sierra Leone (58, 64,028), South Sudan (140, 96,959), Tanzania (7, 4903), Togo (18, 11,976), Uganda (74, 48,503), Zambia (5, 2095), Zimbabwe (1, 700)
Eastern Mediterranean	7	Afghanistan (43, 48,878), Djibouti (7, 2516), Jordan (2, 802), Pakistan (18, 14,200), Somalia (227, 237,498), Sudan (136, 109,099), Yemen (2, 816)
European	3	Albania (1, 906), Macedonia (1, 865), Tajikistan (5, 4337)
The Americas	4	Bolivia (3, 1775), Guatemala (2, 625), Haiti (49, 39,764), Nicaragua (2, 1017)
South-East Asia	7	Bangladesh (26, 13,480), India (8, 5182), Indonesia (3, 1749), Myanmar (22, 14,391), Nepal (12, 7650), Sri Lanka (3, 2586), Thailand (2, 1812)
Western Pacific	1	Philippines (12, 6220)

**Fig. 2:**
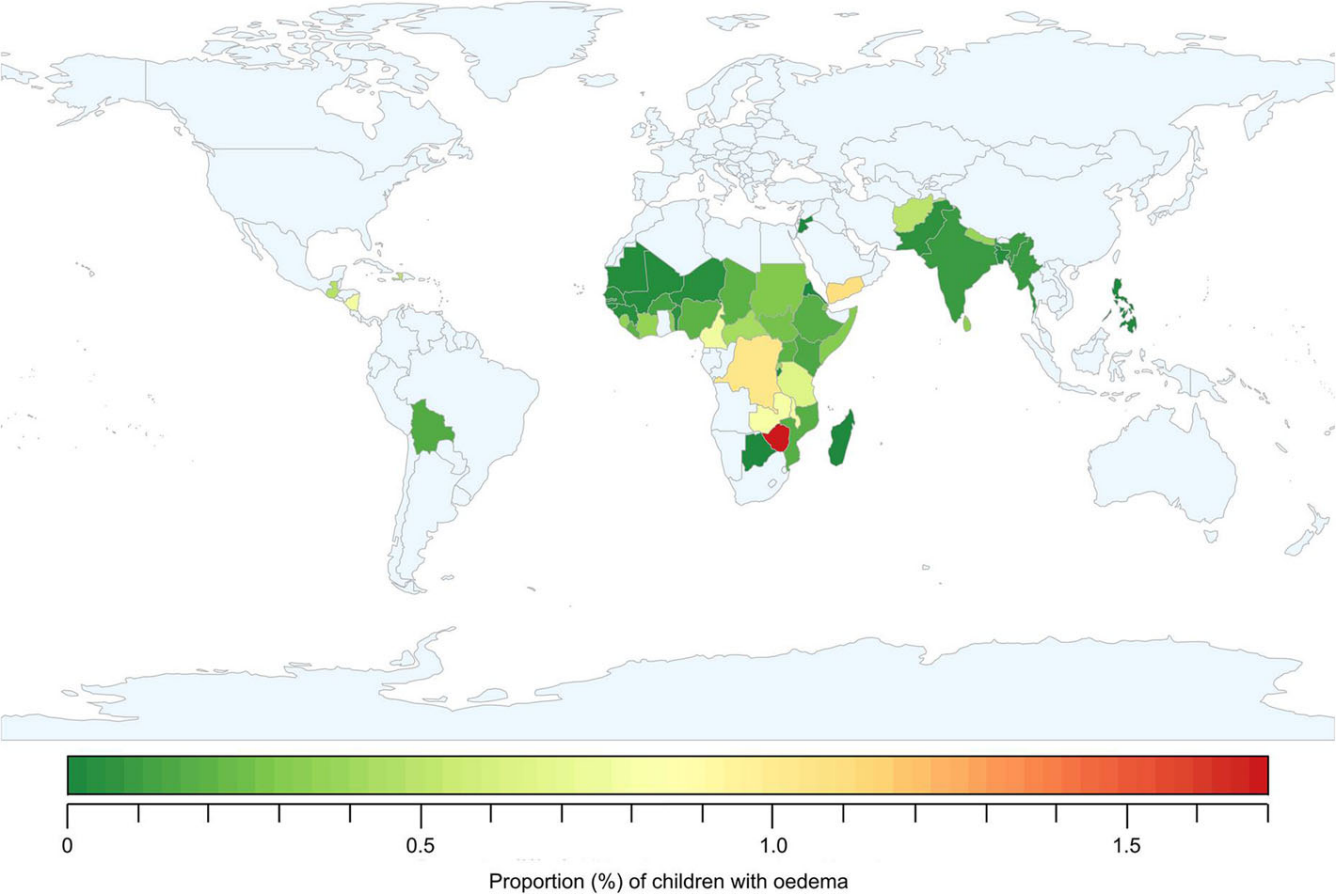
Edema Prevalence according to surveys from 2006 to2015 (excluding countries with < 20 cases of SAM)

Figure 2 shows the prevalence of edema by country as shown by the surveys conducted during the ten-year period from 2006 to 2015. Only countries that reported 20 or more cases of SAM were included in this map. Prevalence was less than 1% in most of the countries where data were available. Some countries in Central and South Africa, as well as Haiti in the Caribbean, reported higher prevalence, and Yemen, Zimbabwe and the Democratic Republic of Congo (DRC) reported prevalence between 1 and 2%.

**Fig. 3:**
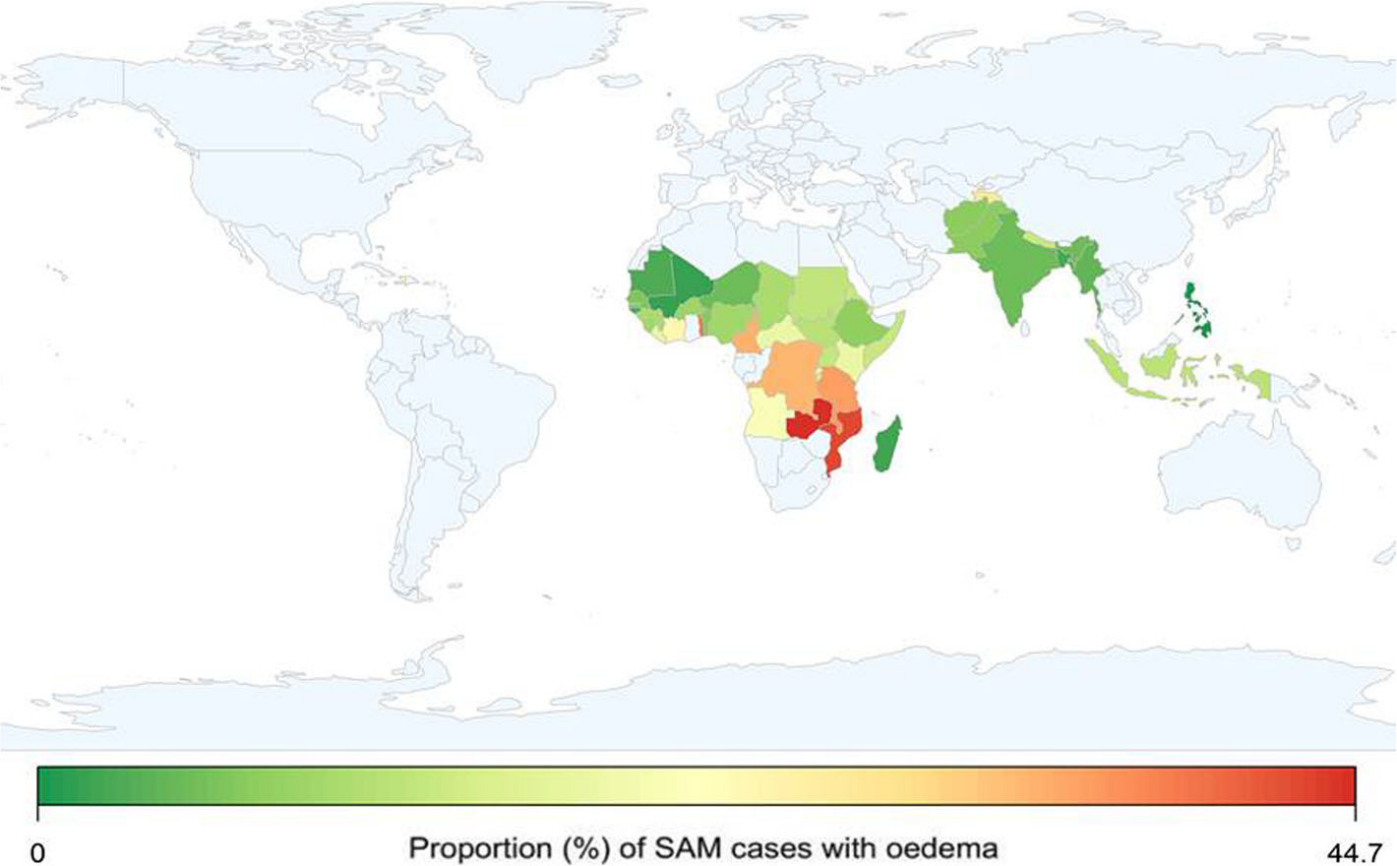
Proportion of SAM cases identified by MUAC with edema (1992–2015, excluding countries with < 20 cases of SAM)

Figure 3 shows the proportion of cases with edema among the SAM cases identified by MUAC < 115 mm only. Countries with less than 20 detected SAM cases were excluded. In order to show the relationship between MUAC and edema, all the datasets were included in this analysis.

According to the data in Fig. 3, surveys from a significant number of countries in Africa indicated that more than a third of SAM cases defined by low MUAC had edema, including Malawi, Rwanda, Zambia, Togo and Cameroon. Notably, the data from Malawi estimated that almost half of all SAM cases with MUAC < 115 mm also had edema.

**Fig. 4:**
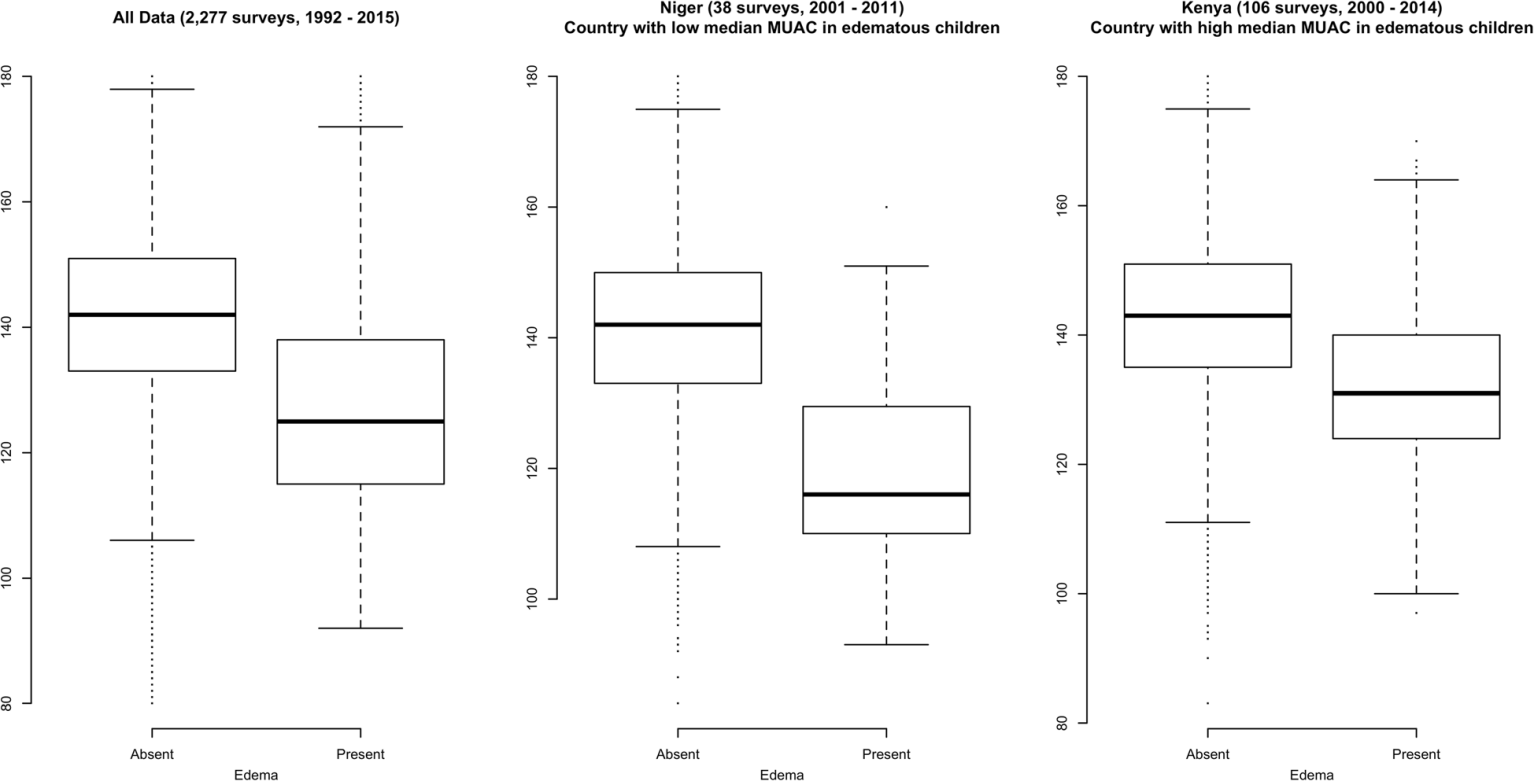
Distribution of MUAC in children with and without edema, 1992–2015 in the whole sample (2277 surveys, 1,725,086 children)

Figure 4 compares the distribution of MUAC between children with and without edema. This figure shows that children with edema tended to have a lower median MUAC (median MUAC = 125 mm) than children without edema (median MUAC = 142 mm). However, it also shows that the MUAC distribution among edematous children has a large spread, with most edematous children (c. 75%) having a MUAC above the 115 mm cut-off used to independently define a child with SAM.

Table 3 shows the median MUAC among children with and without edema, by WHO region and by country.

**Table 3:** Median MUAC among children with and without edema by WHO region and country

		Median and interquartile ranges MUAC per country		Proportion of SAM cases with edema (%)
WHO region	Country	With edema	Without edema	
African Region	Angola	124 (116–136)	140 (130–150)	20.38
	Benin	130 (108–149)	146 (138–155)	6.33
	Burkina Faso	116 (108–130)	143 (135–152)	9.17
	Burundi	130 (120–140)	140 (132–150)	14.13
	Cameroon	130 (120–140)	147 (138–155)	34.02
	Central African Republic	118 (108–127)	143 (135–152)	19.67
	Chad	122 (112–134)	142 (133–150)	10.19
	Congo (Kinshasa)	120 (112–130)	142 (133–151)	32.74
	Ivory Coast	123 (114–134)	147 (138–155)	23.24
	Eritrea	120 (118–130)	140 (130–148)	13.16
	Ethiopia	125 (110–136)	140 (132–147)	7.89
	Gambia	110 (105–115)	147 (139–155)	8.11
	Guinea	122 (114–137)	147 (138–156)	11.01
	Guinea-Bissau	118 (109–128)	149 (141–157)	5.13
	Kenya	131 (124–140)	143 (135–151)	18.05
	Liberia	130 (117–143)	145 (135–155)	18.53
	Madagascar	113 (112–114)	138 (130–146)	1.80
	Malawi	132 (119–144)	144 (135–154)	33.82
	Mali	102 (100–104)	142 (134–151)	1.32
	Mauritania	118 (114–135)	144 (136–153)	3.19
	Mozambique	128 (114–137)	146 (136–154)	41.55
	Niger	116 (110–129)	142 (133–150)	4.86
	Nigeria	117 (108–138)	143 (134–152)	9.57
	Rwanda	130 (1120–140)	141 (132–151)	24.59
	Senegal	112 (108–126)	146 (138–154)	5.88
	Sierra Leone	131 (116–148)	144 (133–154)	9.57
	South Sudan	128 (116–139)	141 (132–150)	11.05
	Tanzania	136 (130–145)	142 (135–151)	34.55
	Togo	146 (125–157)	149 (140–157)	38.89
	Uganda	125 (114–134)	143 (134–152)	12.90
	Zambia	148 (137–159)	148 (139–156)	40.48
	All countries in region	123 (113–135)	142 (134–151)	17.86
Eastern Mediterranean Region	Afghanistan	126 (115–142)	140 (130–149)	7.07
	Djibouti	131 (128–140)	144 (135–153)	14.29
	Pakistan	131 (120–139)	141 (132–150)	6.98
	Somalia	132 (119–144)	141 (133–150)	12.95
	Sudan	136 (124–136)	141 (132–150)	12.08
	All countries in region	132 (120–145)	141 (132–150)	11.56
European Region	Tajikistan	124 (118–136)	140 (130–150)	26.27
Region of the Americas	Haiti	122 (114–134)	145 (137–154)	24.58
South-East Asia Region	Bangladesh	148 (135–154)	141 (134–149)	2.59
	India	128 (124–128)	138 (131–146)	4.85
	Indonesia	125 (116–130)	140 (133–148)	11.76
	Myanmar	116 (110–131)	138,130–147)	4.27
	Nepal	127 (119–134)	138 (130–146)	13.36
	All countries in region	126 (116–134)	140 (132–148)	6.61%
Western Pacific Region	Philippines	NA	150 (142–158)	NA^a^

^a^NA: no cases of edema found, indicator could not be calculated

There was a wide variation of median MUACs, with a range of 102 mm (Mali) to 162 mm (Sri Lanka) for children with edema and 138 mm (Madagascar, India, Myanmar and Nepal) to 157 mm (Bolivia) for children without edema. A consistent trend was found across both individual countries and regions, with the median MUAC lower for children with edema than for children without edema, which is similar to the overall results illustrated in Fig. 4.

Table 4 shows the median MUAC in edematous and non-edematous cases by age group and sex. Year-centered age groups commonly used during nutrition surveys were used for this analysis.

**Table 4:** Median MUAC by age group and sex with 95% confidence intervals

Age group	Number of children with SAM	Percentage of SAM children with edema	Median MUAC (mm) per group	
			With edema	Without edema
Males				
6–17 months	9819	8.78 (8.22–9.34)	120 (119–121)	136 (135–137)
18–29 months	4903	22.33 (21.17–23.50)	125 (124–126)	140 (139–141)
30–41 months	1965	36.54 (34.41–38.67)	131 (129–133)	145 (144–146)
42–53 months	1047	45.56 (42.54–48.58)	135 (133–137)	148 (147–149)
54–59 months	427	50.35 (45.61–55.09)	134 (131–137)	148 (146–149)
Total Males	18,161	18.54 (17.97–19.10)	126 (125–126)	143 (142–144)
Females				
6–17 months	12,625	7.64 (7.18–8.11)	120 (119–121)	133 (132–134)
18–29 months	5993	17.35 (16.39–18.31)	125 (124–126)	139 (138–140)
30–41 months	2200	32.73 (30.77–34.69)	130 (128–132)	145 (144–146)
42–53 months	1029	38.00 (35.03–4.96)	132 (130–134)	148 (147–149)
54–59 months	433	44.34 (39.66–49.02)	140 (137–143)	148 (148–150)
Total Females	22,280	14.85 (14.38–15.31)	125 (124–126)	142 (142–143)

Although SAM by MUAC is more prevalent in younger age groups, the proportion of SAM children with edema is higher for older groups (increasingly from 6 to 17 months to 54–59 months). This was the case for both males and females, with a Chi-Square test for trend as 1848.4 (*p* < 0.0001) and 1795.8 (p < 0.0001) respectively. Median MUAC is lower for children with edema than for children without edema in all age groups, which is consistent with the results shown in Fig. 4 and Table 3.

Figure 4 also shows the MUAC distribution of two countries with very different median MUACs for children with edema. In Niger, the median MUAC of children with edema present was 116 mm, compared with 142 mm for children with edema absent. In contrast, in Kenya, the median MUAC of children with edema averaged 131 mm, compared to 143 mm for children without edema.

## Discussion

**Fig. 5:**
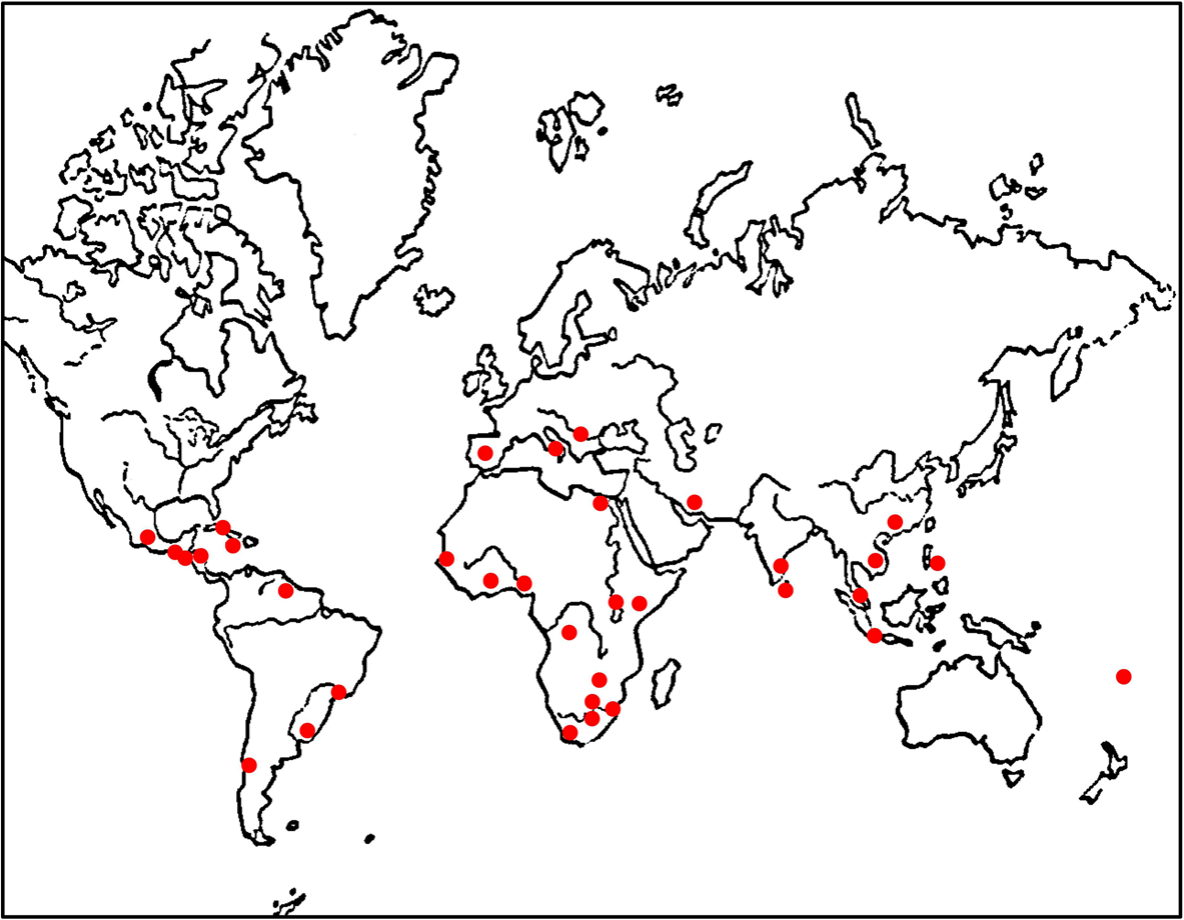
Global map of kwashiorkor as shown by Brock (1954)

To our knowledge, the last published global map showing the occurrence of kwashiorkor was produced in 1954 [17] and was done at a time when the diagnostic criteria were less well defined. It is unclear how this map was obtained, and in the absence of reference to community surveys, it may have been based on hospital records alone as was common at the time. An ecological analysis comparing the distribution of kwashiorkor and liver disease (primary carcinoma and cirrhosis) was presented, and the map below (Fig. 5) is the one shown in the article. The red dots show where cases of kwashiorkor were found.

The present study provides the geographical distribution of edema, based on recent surveys and utilizing more specific, measurable and evidence-based diagnostic criteria.

The findings demonstrate that edema is a common form of SAM, mainly occurring in Central Africa. The associated nutritional status, as assessed by MUAC, shows strong variation among children with edema. This has implications in terms of prognosis, as children with both low MUAC and edema are likely to have a higher risk of death [18–20]. This also has implications for community screening, given that children with edema and high MUAC could potentially be missed if workers do not consistently check for the presence of edema.

The treatment of SAM recommended by WHO is the same for children with or without edema. However, the large variation of MUAC associated with edema suggests very different clinical situations in various settings, most likely with differing nutritional and other therapeutic requirements. It seems plausible that the phase of intensive feeding to promote catch-up growth does not have the same importance in settings where edematous children have a low or a high mean MUAC. In some countries, low MUAC and edema often occur simultaneously, which indicates a large group of extremely high-risk children that may require special, tailored treatment for SAM.

The etiology of edema is still being debated [7]. The presence of children with edema in areas where the background level of malnutrition as assessed by MUAC is low may help distinguish the factors leading to edema development, as opposed to those associated with other types of malnutrition. This should be explored in future studies.

The limitations within this study included that in some countries the number of surveys conducted and children represented were limited. Only 3 European and 4 American countries were represented, presumably due to the low prevalence of SAM in those regions. Furthermore, surveys are usually conducted in areas with suspected nutritional problems, so they are not necessarily representative of the whole country. Therefore, this study cannot be regarded as giving a completely representative picture of the geographical distribution of edematous malnutrition. However, the authors consider that the variation of MUAC associated with edema is likely to reflect a real phenomenon, as the variation cannot be influenced by selection bias.

To date, data on edematous malnutrition have been limited and of varied quality, despite the high mortality related to this disease. It is also thought that there are many more children with edematous malnutrition than suggested by prevalence surveys, as noted more than 40 years ago by Cicely Williams who highlighted the limitations in methodologies for assessing the burden of this acute condition [21]. In surveys and in many countries, edema cases pose a heavy burden to the health care system. The lack of solid data suggests that a more systematic and standardized system for data collection is warranted, to assist both practitioners and researchers.

## Conclusion

There is a critical need for more studies to examine kwashiorkor and for improved data collection on edema within nutritional surveys, SAM management programs and community work to better understand the etiology and prevalence of edematous malnutrition. This would help leverage greater resource prioritization for the identification and prevention of edematous malnutrition, which may include mobilization of governments and donors to provide appropriate support for screening, referral and treatment of children with this life-threatening yet curable condition.

## Abbreviations

ACF: Action Contre la Faim
CMAM: Community-based management of acute malnutrition
DHS: Demographic and health surveys
HAZ: Height-for-age z-score
MAM: Moderate acute malnutrition
MICS: Multiple indicator cluster survey
MUAC: Mid-upper arm circumference
NGO: Non-governmental organisation
SAM: Severe acute malnutrition
SMART: Standardized monitoring and assessment of relief and transitions
UNICEF: United Nations Children’s Fund
WHZ: Weight-for-height z-score
WHO: World Health Organization
